# Exocrine Pancreatic Insufficiency in Diabetes: Association with Cardiovascular Disease and Insulin Therapy

**DOI:** 10.3390/jcm15114319

**Published:** 2026-06-03

**Authors:** Melek Balamir, Bartu Avcı, Elara Coşan, Sinan Tanyolaç, Bilger Çavuş, Aslı Çifcibaşı Örmeci, Filiz Akyüz, Selman Fatih Beşışık, Sabahattin Kaymakoğlu, Hulya Hacısahinogullari, Göktuğ Sarıbeyliler, Ramazan Çakmak, Kubilay Karşıdağ, Şirin Çetin, Kadir Demir

**Affiliations:** 1 Department of Internal Medicine, Istanbul Faculty of Medicine, Istanbul University, Istanbul 34093, Turkey; 2 Istanbul Faculty of Medicine, Istanbul University, Istanbul 34093, Turkey; avcib@upmc.edu (B.A.); elaracosan@gmail.com (E.C.); 3 Division of Endocrinology and Metabolism, Department of Internal Medicine, Istanbul Training and Research Hospital, Istanbul 34098, Turkey; stanyolac@gmail.com; 4 Division of Gastroenterohepatology, Department of Internal Medicine, Istanbul Faculty of Medicine, Istanbul University, Istanbul 34093, Turkey; dr_bilgercavus@yahoo.com (B.Ç.); aslic79@yahoo.com.tr (A.Ç.Ö.); filizakyuz@hotmail.com (F.A.); besisiksef@yahoo.com (S.F.B.); kaymakoglus@hotmail.com (S.K.); kadirdmr@yahoo.com (K.D.); 5 Division of Endocrinology and Metabolism, Department of Internal Medicine, Istanbul Faculty of Medicine, Istanbul University, Istanbul 34093, Turkey; mercandogru@hotmail.com (H.H.); saribeyliler@gmail.com (G.S.); rmznckmk@yahoo.com (R.Ç.); kubilaykarsidag@yahoo.com (K.K.); 6 Department of Biostatistics, Faculty of Medicine, Amasya University, Amasya 05100, Turkey; cetinsirin55@gmail.com

**Keywords:** diabetes mellitus, exocrine pancreatic insufficiency, fecal elastase-1, insulin therapy, cardiovascular disease

## Abstract

**Background/Objectives**: Exocrine pancreatic insufficiency (EPI) is increasingly recognized in patients with diabetes; however, its clinical correlates remain poorly defined. This study aimed to determine the prevalence and clinical characteristics of EPI in patients with type 1 (T1DM) and type 2 diabetes mellitus (T2DM) and to evaluate its associations with diabetes-related complications and insulin therapy. **Methods**: A total of 200 patients with diabetes were screened, and 182 who met the inclusion criteria were included in the final analysis. EPI was diagnosed using fecal elastase-1 (FE-1). Clinical, biochemical, and complication-related data were collected. Factors associated with EPI were evaluated using univariate and multivariate logistic regression analyses. Among patients with T2DM, an inverse probability of treatment weighting–average treatment effect on the treated (IPTW-ATT) model was constructed to evaluate the association between insulin therapy and EPI. Propensity scores were estimated using baseline demographic and clinical covariates, and covariate balance after weighting was assessed using standardized mean differences (SMDs). **Results**: Exocrine pancreatic insufficiency was detected in 18.1% of patients, with prevalences of 21.2% in T1DM and 17.4% in T2DM. Cardiovascular disease was the only variable independently associated with EPI in multivariate analysis (OR = 3.25; 95% CI: 1.12–6.75; *p* = 0.028. Among patients with T2DM, insulin therapy was significantly associated with EPI in both unadjusted and IPTW-ATT analyses (weighted OR = 10.76; 95% CI: 1.85–62.76; *p* = 0.008) with a wide confidence interval reflecting sparse data. Cardiovascular disease also remained significantly associated with EPI in the weighted model (OR = 3.52; 95% CI: 1.22–10.15; *p* = 0.020). **Conclusions**: Exocrine pancreatic insufficiency is a clinically relevant condition in diabetes and shows a significant cross-sectional association with cardiovascular disease. In T2DM, insulin therapy was associated with a higher prevalence of EPI, although confounding by indication cannot be excluded. These findings suggest that evaluation of exocrine pancreatic function may be considered in high-risk diabetic subgroups, pending confirmation in prospective longitudinal studies.

## 1. Introduction

Exocrine pancreatic insufficiency (EPI) results from reduced pancreatic enzyme secretion or activity, leading to maldigestion, particularly of fats [1,2]. Although the association between type 3c (pancreatogenic) diabetes and pancreatic disorders such as chronic pancreatitis, pancreatic cancer-related ductal obstruction, pancreatic surgery, and cystic fibrosis is well established, the impact of diabetes itself on exocrine pancreatic function remains incompletely understood [3,4]. In type 1 diabetes mellitus (T1DM), loss of the trophic effects of insulin, autoimmune pancreatic injury, autonomic neuropathy with disruption of the entero-pancreatic reflex, and microvascular damage may contribute to exocrine dysfunction. In type 2 diabetes mellitus (T2DM), autonomic neuropathy and microvascular injury may promote pancreatic atrophy and fibrosis, particularly in individuals with marked insulin resistance [5,6]. Although EPI is present in nearly all patients with type 3c diabetes, increasing attention has recently focused on the prevalence and clinical significance of EPI in patients with T1DM and T2DM. Measurement of FE-1 is widely used to assess exocrine pancreatic function, as the enzyme remains stable during intestinal transit [2]. FE-1 testing is non-invasive and does not require timed stool collection or specific dietary restrictions [7]. Søfteland et al. defined EPI as a fecal elastase-1 (FE-1) concentration ≤ 200 μg/g stool and reported a prevalence of 12.7% among diabetic patients, with higher rates in T1DM than in T2DM [8].

This study aimed to evaluate the prevalence and clinical characteristics of EPI in patients with T1DM and T2DM and to investigate its association with microvascular and macrovascular complications.

## 2. Materials and Methods

### 2.1. Study Design and Setting

This single-center, cross-sectional study was conducted in the endocrinology and gastroenterohepatology divisions of Istanbul University, Istanbul Faculty of Medicine, a tertiary referral center. Diabetes type was determined at the time of initial diagnosis during routine clinical evaluation, including assessment of autoimmune markers (anti-GAD and anti-IA2 antibodies), and only patients with a confirmed diagnosis of T1DM or T2DM were included. A total of 200 patients aged ≥18 years with a diagnosis of T1DM or T2DM for at least 5 years were initially screened. Written informed consent was obtained from all participants. Eighteen patients were excluded because of pregnancy or breastfeeding, incomplete medical records, chronic alcohol use, or known pancreatic disease, including chronic pancreatitis, pancreatic cancer, or prior pancreatic surgery, leaving 182 patients for the final analysis.

Laboratory parameters, including serum amylase, lipase, fasting and postprandial plasma glucose, fasting insulin, C-peptide, hemoglobin A1c (HbA1c), alanine aminotransferase (ALT), aspartate aminotransferase (AST), alkaline phosphatase (ALP), gamma-glutamyl transferase (GGT), blood urea nitrogen (BUN), creatinine, calcium, phosphorus, 25-hydroxyvitamin D3 (25-OHD3), low-density lipoprotein cholesterol (LDL-C), high-density lipoprotein cholesterol (HDL-C), and triglycerides (TG), were retrieved from patient records. According to the American Diabetes Association guidelines, target glycemic control was defined as an HbA1c level < 7%, whereas values ≥ 7% were considered indicative of poor glycemic control [9]. Body mass index (BMI), smoking status, and antidiabetic medications were recorded; antidiabetic therapy included insulin, metformin, sulfonylureas, and dipeptidyl peptidase-4 inhibitors. Diabetic complications were assessed using patient records. Retinopathy was evaluated by an ophthalmologist, nephropathy was defined based on the presence of microalbuminuria, and neuropathy was assessed clinically, with monofilament testing and electromyography performed when peripheral neuropathy was suspected. Patients with diabetes underwent routine follow-up for diabetes-related complications, including ophthalmologic and cardiologic evaluations. Cardiology assessments typically included electrocardiography (ECG) and echocardiography, with additional investigations, such as stress testing or coronary angiography, performed when clinically indicated. Cardiovascular disease was defined based on angiographic findings in patients who underwent coronary angiography for cardiac symptoms, as documented in the medical records. Among the 182 patients included in the study, 62 underwent coronary angiography, and 43 were classified as having cardiovascular disease based on angiographic findings considered clinically significant by the treating cardiologist. A standardized angiographic stenosis threshold was not predefined in the study protocol. Patients without angiographically documented clinically significant coronary artery disease were classified as not having cardiovascular disease for the purposes of the present analysis. Peripheral vascular disease was identified based on reduced, absent or asymmetric peripheral pulses on physical examination and was confirmed by arterial Doppler ultrasonography when abnormal findings or symptoms such as intermittent claudication, were present. Patient-reported gastrointestinal symptoms, including diarrhea, steatorrhea, abdominal pain, bloating, and weight loss, were assessed through direct clinical questioning.

Fecal elastase-1 (FE-1) levels were measured in stool samples obtained from all eligible patients using a sandwich enzyme-linked immunosorbent assay (ELISA) (Bioserv Diagnostics GmbH, Rostock, Germany)with two monoclonal antibodies specific for pancreatic elastase. No specific dietary instructions were provided before sample collection. Stool samples were stored at −80 °C and analyzed weekly. According to the manufacturer’s instructions, FE-1 remains stable in frozen samples for up to 28 days. Patients were classified as having normal exocrine pancreatic function (FE-1 ≥ 200 μg/g), mild-to-moderate EPI (FE-1 100–199 μg/g), or severe EPI (FE-1 < 100 μg/g) [10].

The study was conducted in accordance with the Declaration of Helsinki and was approved by the Clinical Research Ethics Committee of Istanbul University, Istanbul Faculty of Medicine (File No: 2017/426; Approval No: 443; Date: 24 April 2017). In 2019, a subsequent application with a minor revision to the study title was submitted under the same file number and approved with Approval No: 459 on 29 March 2019. However, the study itself was based on the original ethical approval granted in 2017.

### 2.2. Statistical Analysis

Statistical analyses were performed using SPSS version 20.0 (SPSS Inc., Chicago, IL, USA), and a two-sided *p*-value < 0.05 was considered statistically significant. Categorical variables were expressed as frequencies and percentages, whereas continuous variables were presented as mean ± standard deviation (SD). The Shapiro–Wilk test was used to assess the normality of data distribution. Comparisons between two groups were performed using Student’s *t*-test or the Mann–Whitney U test, as appropriate. Correlations were assessed using Pearson’s or Spearman’s correlation coefficients. Comparisons among more than two groups were performed using one-way analysis of variance (ANOVA) or the Kruskal–Wallis test, as appropriate. Associations between categorical variables were evaluated using the chi-square test or Fisher’s exact test. A post hoc power analysis was performed using G*Power software (version 3.1.9.2) to assess the statistical power of the comparison of EPI prevalence between the T1DM and T2DM subgroups. Univariate and multivariate logistic regression analyses were subsequently performed to identify factors associated with the presence of EPI. For the multivariate logistic regression model, variables were selected based on either a *p*-value < 0.25 in univariate analysis or a priori clinical relevance. Backward elimination was applied with a retention criterion of *p* < 0.10, while clinically relevant variables were retained regardless of statistical significance. Additionally, among patients with T2DM (*n* = 149), an inverse probability of treatment weighting–average treatment effect on the treated (IPTW–ATT) logistic regression model was constructed to evaluate the association between insulin therapy and EPI. The IPTW-ATT analysis was restricted a priori to patients with T2DM because all patients with T1DM (*n* = 33, 100%) were receiving insulin therapy, resulting in complete separation and precluding reliable propensity score estimation. When the treatment variable shows no within-group variation, the logistic regression model used for treatment assignment cannot converge, resulting in degenerate propensity scores (*p* = 1.0 for all subjects). Therefore, this structural limitation—rather than a post hoc analytical decision—necessitated restricting the IPTW analysis to patients with T2DM, among whom both insulin-treated (*n* = 106, 71.1%) and non-insulin-treated (*n* = 43, 28.9%) individuals were available for comparison. Propensity scores for insulin therapy were estimated using logistic regression based on the following baseline covariates: age, sex, BMI, HbA1c, hypertension, hyperlipidemia, weight, height, and cardiovascular disease. Missing data rates for baseline covariates included in the propensity score model were assessed prior to analysis. HDL-C and LDL-C had substantial missing data rates (42.3% and 36.2%, respectively) and were therefore excluded from the propensity score model to avoid introducing bias through extensive imputation or case deletion. Hypertension (8.1%) and hyperlipidemia (6.0%) had low missing data rates and were imputed using the mode. All remaining covariates (age, sex, BMI, HbA1c, weight, height, and cardiovascular disease) had complete data (0% missing). Covariate balance before and after weighting was assessed using standardized mean differences (SMDs), with absolute SMD values < 0.10 considered indicative of excellent balance and values < 0.20 considered acceptable. Model discrimination was evaluated using the weighted area under the receiver operating characteristic curve (AUC). As a sensitivity analysis, the IPTW-ATT model was re-estimated using a reduced covariate set including age, sex, BMI, HbA1c, and cardiovascular disease.

## 3. Results

The demographic and clinical characteristics of the study population are summarized in Table 1. A total of 182 patients were included in the final analysis, of whom 18.1% (*n* = 33) had T1DM and 81.9% (*n* = 149) had T2DM. The mean age was 55.7 ± 12.9 years (range: 22–84), and 40.1% (*n* = 73) of participants were male. As shown in Table 1, patients with T2DM were older and had higher BMI values, whereas patients with T1DM had a longer duration of diabetes. Diabetic complications were common overall, with neuropathy being the most prevalent.

When stratified according to EPI status, cardiovascular disease was significantly more frequent among patients with EPI than among those without EPI (*p* = 0.020). In contrast, diabetes duration, other diabetic complications, and peripheral vascular disease were comparable between the groups. Gastrointestinal symptoms were more common in patients with EPI; however, the difference did not reach statistical significance.

The prevalence and subgroup distribution of exocrine pancreatic insufficiency are summarized in Table 2. EPI was detected in 18.1% (*n* = 33) of the total study population, with a prevalence of 21.2% in patients with T1DM and 17.4% in those with T2DM, with no significant difference between diabetes types (*p* = 0.780). However, given the observed sample sizes (*n* = 33 for T1DM and *n* = 149 for T2DM), an alpha level of 0.05, and the observed EPI prevalences of 21.2% and 17.4%, respectively, the statistical power was estimated at approximately 7%. Mild–moderate EPI was more common than severe EPI, accounting for 11.5% and 6.6% of cases, respectively. The distribution of EPI categories did not differ significantly between diabetes types.

The prevalence of at least one gastrointestinal symptom was 53.8% (*n* = 98) in the entire cohort and 63.6% (*n* = 21) among patients with EPI; however, this difference was not statistically significant (*p* = 0.25).

The results of the univariate and multivariate logistic regression analyses are presented in Table 3. In univariate analysis, cardiovascular disease was significantly associated with EPI (OR = 2.58; 95% CI: 1.15–5.77; *p* = 0.021), whereas no other variables reached statistical significance. In the multivariate logistic regression model, cardiovascular disease remained significantly associated with EPI (OR = 3.25; 95% CI: 1.12–6.75; *p* = 0.028).

Laboratory parameters are summarized in Table 4. FE-1 levels were significantly lower in patients with EPI than in those without EPI (*p* < 0.001). Among the remaining laboratory variables, only calcium levels were significantly lower in patients with EPI (*p* = 0.043), whereas no other significant differences were observed.

Correlation analysis demonstrated weak negative correlations between FE-1 levels and both ALP (r = −0.167, *p* = 0.024) and creatinine levels (r = −0.206, *p* = 0.005), whereas no significant correlations were observed with other clinical or biochemical parameters.

Achievement of target HbA1c levels did not differ between patients with and without EPI in either T1DM or T2DM subgroups.

Among T2DM patients, insulin use was more frequent in those with EPI compared with those without EPI (88.5% vs. 65.0%, *p* = 0.020) (Table 5).

In the IPTW-ATT weighted logistic regression model (AUC = 0.769), insulin therapy was significantly associated with EPI (OR = 10.76; 95% CI: 1.85–62.76; *p* = 0.008). The wide confidence interval likely reflects the limited number of EPI events and the sparse data in the non-insulin-treated EPI subgroup (*n* = 3). In a sensitivity analysis using a reduced covariate set (age, sex, BMI, HbA1c, and cardiovascular disease), the association remained directionally consistent (OR = 11.14; 95% CI: 1.85–67.10; *p* = 0.009). Cardiovascular disease also remained significantly associated with EPI (OR = 3.52; 95% CI: 1.22–10.15; *p* = 0.020). No other covariates reached statistical significance (Table 6).

After IPTW-ATT weighting, three of nine covariates achieved excellent balance (SMD < 0.10: age, hypertension, and hyperlipidemia) and three additional covariates showed acceptable balance (SMD 0.10–0.20: sex, BMI, and cardiovascular disease). Residual imbalance was observed for HbA1c (SMD = 0.25), weight (SMD = 0.22), and height (SMD = 0.22). Notably, the HbA1c imbalance exceeded both the conventional threshold of 0.10 and the predefined acceptable threshold of 0.20 (Table 7).

## 4. Discussion

Exocrine pancreatic insufficiency is relatively common in patients with diabetes; however, its relationship with diabetes remains incompletely understood. Reported prevalence rates vary substantially across studies, likely reflecting heterogeneity in diagnostic methods and patient populations. In our study population, EPI was identified in 18.1% of diabetic patients (21.2% in T1DM and 17.4% in T2DM). Cardiovascular disease was more common among patients with EPI and was the only variable independently associated with EPI in multivariate analysis. In the T2DM subgroup, EPI was more frequent among patients receiving insulin therapy, and this association remained significant after IPTW-ATT adjustment.

The reported prevalence of EPI varies substantially across the literature. A recent review reported prevalence rates ranging from 14% to 77.5% in T1DM and from 16.8% to 49.2% in T2DM. In studies in which diabetes type was not specified, prevalence estimates showed even greater variability, ranging from 5.4% to 77%. In non-diabetic control populations, the reported prevalence of EPI ranged from 4.4% to 18% (median, 13%) [11]. In 2013, Vujasinovic et al. reported a relatively low EPI prevalence of 5.4% in a cohort of 150 patients with diabetes, including 50 patients with T1DM and two T2DM subgroups stratified according to insulin use. The authors attributed this lower prevalence to their strict exclusion criteria, which excluded patients with diabetes duration < 5 years as well as those with coexisting pancreatic, hepatic, malignant, inflammatory, autoimmune, or other gastrointestinal disorders [12]. In 2016, Demir et al. evaluated 216 patients with diabetes, including 70 patients with T1DM, all of whom had a diabetes duration of more than 5 years, and reported an overall EPI prevalence of 14.4%, including 15.7% in patients with T1DM and 13.7% in those with T2DM. The authors attributed the lower prevalence observed compared with earlier studies to the more widespread use of FE-1 testing [13]. More recently, a meta-analysis by Zhang et al. reported a pooled EPI prevalence of 22% (95% CI: 15–31%) among patients with T2DM. The authors further noted that EPI prevalence was higher in Asian countries (35%; 95% CI: 22–49%) than in Europe (18%; 95% CI: 10–29%) or Australia (9%; 95% CI: 4–16%) [14]. The prevalence of EPI observed in our study was comparable to the lower range of estimates reported in European cohorts. This relatively low prevalence may be attributable to our stringent exclusion criteria, including exclusion of patients with diabetes duration < 5 years, pregnancy, a history of pancreatitis, or chronic alcohol consumption, as well as the use of FE-1 as a standardized diagnostic tool.

A meta-analysis of 40 studies reported pooled EPI prevalences of 40% (26–74%) in T1DM and 27% (10–56%) in T2DM [15]. However, more recent studies using FE-1 testing and stricter exclusion criteria have reported smaller differences between diabetes types. Consistent with these findings, we likewise observed no significant difference in EPI prevalence between patients with T1DM and T2DM. Nevertheless, this comparison was severely underpowered and should not be interpreted as evidence of equivalence; therefore, findings related to the T1DM subgroup should be considered exploratory.

In addition, methodological considerations related to FE-1 measurement should be taken into account when interpreting these prevalence estimates. FE-1 is a widely used, non-invasive test for the assessment of exocrine pancreatic function and represents a practical screening tool with a high negative predictive value for excluding EPI [7]. However, as an indirect measure, its diagnostic performance may be influenced by factors unrelated to pancreatic function. Because FE-1 is measured as a stool concentration, dilution in patients with watery stools may result in falsely low values and potential overestimation of EPI, whereas its limited sensitivity in mild-to-moderate EPI may contribute to underdiagnosis [1]. In our study, all stool samples were formed (Bristol Stool Form Scale types 4–5), and no patients had watery stools (types 6–7) or clinically evident diabetic diarrhea, making dilution-related bias unlikely to have substantially affected our findings.

Weak negative correlations were observed between FE-1 levels and both ALP and creatinine. Elevated ALP levels may reflect hepatobiliary or bone-related processes, whereas higher creatinine levels may be associated with renal dysfunction in the context of diabetic microvascular disease. However, given the weak magnitude of these correlations and the absence of mechanistic evaluation, these findings should be interpreted cautiously and regarded as hypothesis-generating.

Diabetes is frequently accompanied by microvascular, macrovascular, and autonomic complications that may impair exocrine pancreatic function through mechanisms such as reduced perfusion, autonomic dysfunction, or chronic inflammation. Nevertheless, the relationship between EPI and diabetes-related complications remains incompletely understood. In 2019, de la Iglesia et al. demonstrated that EPI was associated with a higher incidence of cardiovascular events even in the absence of diabetes (incidence rate ratio, 3.67; 95% CI: 1.92–7.24; *p* < 0.001), and that this risk increased further when EPI coexisted with diabetes (EPI without diabetes: OR, 4.96; 95% CI: 1.68–14.65; EPI with diabetes: OR, 6.54; 95% CI: 2.71–15.77) [16]. Similarly, a meta-analysis of 15 studies demonstrated that cardiac autonomic neuropathy was associated with a 3.65-fold increase in overall mortality among patients with diabetes (95% CI: 2.66–4.47), likely mediated by QT prolongation-related malignant arrhythmias, silent myocardial ischemia, impaired heart rate variability, and hemodynamic instability [17]. In addition, Sangnes et al. reported that patients with diabetes and EPI exhibited reduced heart rate variability and impaired baroreflex sensitivity, further supporting autonomic dysfunction as a potential contributing mechanism [18]. These findings suggest that malnutrition-related vascular impairment and autonomic dysfunction may be associated with the increased cardiovascular risk observed in diabetic patients with EPI. Conversely, hemodynamic disturbances, chronic systemic inflammation, and diabetes-related angiopathic changes associated with cardiovascular disease may impair pancreatic perfusion and promote fibrotic remodeling, thereby potentially exacerbating exocrine pancreatic dysfunction. Our findings support this association; however, because coronary angiography was performed only in patients with cardiac symptoms or other clinical indications, asymptomatic or subclinical coronary artery disease may have remained undetected, particularly in patients with T2DM, in whom silent myocardial ischemia is relatively common [19,20]. Conversely, some symptomatic patients who underwent coronary angiography did not have clinically significant coronary artery disease confirmed by angiography and were therefore classified as not having cardiovascular disease. Consequently, cardiovascular disease ascertainment may have been influenced by underrecognition of subclinical disease and variability in referral and diagnostic practices. In addition, unrecognized coronary artery disease may have been present in both the EPI and non-EPI groups, complicating interpretation of the observed association. Therefore, although misclassification of cardiovascular disease status may have attenuated the observed association between cardiovascular disease and EPI, the overall direction and magnitude of the resulting bias remain uncertain. Large prospective studies incorporating standardized cardiovascular assessment protocols are needed to further clarify the relationship between cardiovascular disease and EPI in patients with diabetes.

Our study also demonstrated that EPI was more prevalent among patients with T2DM requiring insulin therapy for glycemic control. These findings are consistent with those reported by Hardt et al. [6] and Vujasinovic et al. [12]. Moreover, the meta-analysis by Zhang et al. further supports this association by demonstrating that patients with higher insulin requirements—potentially reflecting greater insulin deficiency—had a substantially higher prevalence of EPI (27% vs. 15%) [14]. Higher insulin requirements in patients with T2DM may reflect either increased insulin resistance or more advanced insulin deficiency, both of which may diminish the trophic effects of insulin on pancreatic acinar cells and impair pancreatic enzyme synthesis.

A subset of insulin-requiring patients classified as having T2DM may, in fact, have unrecognized type 3c diabetes [21]. In 2017, Woodmansey et al. reported that most cases of diabetes developing after pancreatic disease were misclassified as T2DM (87.8%), whereas only 2.7% were correctly identified as diabetes of the exocrine pancreas. The authors also demonstrated that insulin use within five years was substantially more frequent in patients with diabetes secondary to chronic pancreatic disease than in those with T2DM (45.8% vs. 4.1%) [22]. In our study, misclassification of type 3c diabetes cannot be excluded and may partially account for the observed insulin–EPI association, as pancreatic imaging was not systematically performed.

Insulin therapy remained significantly associated with EPI after IPTW-ATT adjustment (OR = 10.76; 95% CI: 1.85–62.76; *p* = 0.008). However, the magnitude of this association should be interpreted cautiously, as the wide confidence interval reflects the limited number of EPI events and sparse data in the non-insulin-treated EPI subgroup. Furthermore, the observed association is likely influenced by confounding by indication. Patients requiring insulin therapy typically have longer disease duration, more advanced beta-cell dysfunction, and a greater burden of diabetic complications, all of which may independently contribute to exocrine pancreatic dysfunction. Although IPTW-ATT analysis was performed to mitigate this confounding and the association remained significant, residual confounding cannot be excluded in a cross-sectional study design.

The persistent HbA1c imbalance after IPTW-ATT weighting (SMD = 0.25) suggests that confounding by indication was not fully mitigated by the propensity score model, given that HbA1c is a key clinical determinant of insulin therapy assignment. Although sensitivity analysis using a reduced covariate set yielded a directionally consistent and statistically significant result (OR = 11.14; 95% CI: 1.85–67.10; *p* = 0.009), supporting the robustness of the observed association, residual confounding related to HbA1c may still have influenced the magnitude of the insulin–EPI association. Therefore, this estimate should be interpreted as reflecting a strong association rather than a precise effect size.

The high rates of missing data for HDL-C (42.3%) and LDL-C (36.2%) also warrant consideration when interpreting the IPTW-ATT findings. The high missingness likely reflects incomplete lipid panel ordering in routine clinical practice throughout the study period. The missingness pattern was considered most consistent with missing at random (MAR), as lipid testing was probably driven by clinically observed factors such as hyperlipidemia, cardiovascular risk profile, or specialist referral patterns, rather than by the lipid values themselves. However, missing not at random (MNAR) cannot be entirely excluded, as clinicians may have been less likely to order lipid panels in patients perceived to be at lower cardiovascular risk, potentially resulting in systematic underrepresentation of patients with normal lipid profiles. Because HDL-C and LDL-C were excluded from the propensity score model due to this high missingness, residual confounding by lipid-related factors cannot be ruled out. This is particularly relevant because these variables may be associated with both insulin therapy assignment and cardiovascular disease burden, which are the two main exposures evaluated in this study. Therefore, the insulin–EPI association should be regarded as hypothesis-generating rather than causal and requires confirmation in prospective longitudinal studies.

### 4.1. Clinical Implications of EPI in Diabetes

Previous studies suggest that gastrointestinal symptoms are more frequent in diabetic patients with EPI, whereas classical manifestations such as steatorrhea and weight loss may not represent the predominant clinical features in this population [23,24]. Given that nearly one in five patients with diabetes in our cohort had EPI, recognition of these clinical manifestations may have important implications for nutritional status, glycemic stability, and overall quality of life. Although our findings are consistent with the existing literature, the absence of a significant difference between groups may reflect the predominance of mild-to-moderate EPI and the nonspecific nature of gastrointestinal symptoms in patients with diabetes. The relatively small number of EPI cases may also have limited the statistical power of these analyses. Beyond gastrointestinal symptoms, EPI may contribute to maldigestion, nutritional deficiencies, impaired bone health, and reduced quality of life [25]. In our study population, only calcium levels were significantly lower in patients with EPI, whereas broader nutritional consequences could not be comprehensively evaluated.

Another clinically relevant consideration is the potential association between EPI and unexplained hypoglycemia, particularly in long-standing insulin-treated diabetes. Impaired digestion and nutrient malabsorption may contribute to glycemic instability. Previous studies have reported a higher frequency of hypoglycemia in patients with severe FE-1 deficiency [26], whereas pancreatic enzyme replacement therapy (PERT) has been associated with improved glycemic variability in some studies [15,23]. Therefore, FE-1 testing may be considered in selected patients with diabetes who present with unexplained hypoglycemia after more common causes have been excluded. Although PERT use was not systematically evaluated in our cohort, it may offer clinical benefit in patients with EPI and glycemic instability.

### 4.2. Strengths of the Study

1. This study is among the few to evaluate not only the prevalence of EPI in diabetes but also its associations with diabetic complications and insulin therapy, thereby providing clinically relevant insights beyond prevalence estimates alone.
2. Strict exclusion criteria were applied to minimize heterogeneity and reduce potential confounding from conditions known to affect exocrine pancreatic function.
3. The inclusion of patients with long-standing diabetes allowed for a more accurate evaluation of the chronic effects of diabetes on exocrine pancreatic function.

### 4.3. Limitations of the Study

1. The cross-sectional design precludes causal inference and limits the ability to exclude residual confounding, reverse causation, and shared disease-related mechanisms.
2. The unequal distribution of T1DM and T2DM cases, together with the small number of patients in the severe EPI subgroup, limited the robustness of subgroup analyses and ordinal logistic regression models. In addition, IPTW-ATT analysis could not be performed in patients with T1DM because all patients with T1DM were receiving insulin therapy, precluding propensity score estimation and limiting the generalizability of the observed insulin–EPI association to T1DM.
3. The IPTW-ATT analysis was based on a limited number of EPI events among patients with T2DM and substantial imbalance in insulin exposure among EPI cases, which may have reduced model stability, widened confidence intervals, and limited the precision of the estimated insulin–EPI association.
4. High missingness in HDL-C and LDL-C, together with persistent HbA1c imbalance after IPTW-ATT weighting, may have contributed to residual confounding in the IPTW-ATT analysis.
5. The absence of a healthy control group and the relatively modest sample size may limit the generalizability of the findings.
6. The use of FE-1 as the sole diagnostic modality may have introduced misclassification bias because of its limited sensitivity in mild-to-moderate EPI and its susceptibility to non-pancreatic factors such as stool consistency.
7. Cardiovascular disease was defined based on angiographic findings in symptomatic patients, which may have resulted in underrecognition of asymptomatic or subclinical disease. Furthermore, the absence of a predefined angiographic stenosis threshold may have introduced misclassification bias.
8. The absence of systematic pancreatic imaging limited the ability to exclude type 3c diabetes, and disease misclassification cannot be ruled out.

## 5. Conclusions

Exocrine pancreatic insufficiency is a clinically relevant condition in diabetes and was significantly associated with cardiovascular disease in our study population. Among patients with T2DM, insulin therapy was associated with a higher prevalence of EPI, although residual confounding cannot be excluded. Given the methodological limitations of the study, these findings should be considered hypothesis-generating and require confirmation in prospective longitudinal studies before implications for targeted evaluation or clinical management can be established.

## Figures and Tables

**Table 1:** Demographic and clinical characteristics of the study population.

	T1DM *n* (%)	T2DM *n* (%)	*p*	Patients Without EPI	Patients with EPI	*p*	Total
Age (year)	38.6 ± 11.8	59.5 ± 9.7	<0.001	56.1 ± 12.7	53.9 ± 13.8	0.390	55.7 ± 12.9
BMI (kg/m^2^)	25.4 ± 5.2	30.9 ± 6.9	<0.001	29.9 ± 7.0	30.1 ± 6.4	0.830	29.9 ± 6.9
Duration of diabetes (year)	19.1 ± 8.4	15.9 ± 7.7	0.040	16.7 ± 8.1	15.6 ± 7.2	0.500	16.6 ± 7.9
Gender (male)	7 (21.2)	66 (44.3)	0.018	58 (38.9)	15 (45.5)	0.550	73 (40.1)
Diabetic complications	16 (48.5)	118 (79.2)	0.001	107 (71.8)	27 (81.8)	0.280	134 (73.6)
Neuropathy	10 (30.3)	79 (53.0)	0.021	69 (46.3)	20 (60.6)	0.170	89 (48.9)
Retinopathy	6 (18.2)	41 (27.5)	0.370	38 (25.5)	9 (27.3)	0.790	47 (25.8)
Cardiovascular disease	1 (3.0)	42 (28.2)	0.001	30 (20.1)	13 (39.4)	0.020	43 (23.6)
Nephropathy	8 (24.2)	46 (30.9)	0.530	41 (27.5)	13 (39.4)	0.200	54 (29.7)
Peripheral vascular disease	1 (3)	22 (14.8)	0.083	17 (11.4)	6 (18.2)	0.382	23 (12.6)
Cerebrovascular disease	0	2 (1.3)	-	1 (0.6)	1 (3.0)	0.330	2 (1.1)
Insulin	33 (100)	103 (69.1)	<0.001	106 (71.1)	30 (90.9)	0.025	136 (74.7)
OAD	5 (15.2)	113 (75.8)	<0.001	105 (70.5)	13 (39.3)	0.001	118 (64.8)

T1DM: type 1 diabetes mellitus; T2DM: type 2 diabetes mellitus; EPI: exocrine pancreatic insufficiency; BMI: body mass index; OAD: oral anti-diabetic drug(s).

**Table 2:** Distribution of exocrine pancreatic insufficiency in the study group.

	T1DM *n* (%)	T2DM *n* (%)	Total *n* (%)	*p*
Normal exocrine function	26 (78.8)	123 (82.6)	149 (81.9)	0.780
Mild–moderate EPI	5 (15.2)	16 (10.7)	21 (11.5)	
Severe EPI	2 (6.0)	10 (6.7)	12 (6.6)	

T1DM: type 1 diabetes mellitus; T2DM: type 2 diabetes mellitus; EPI: exocrine pancreatic insufficiency.

**Table 3:** Univariate and multivariate logistic regression analysis.

Variable	OR	Lower	Upper	*p*	OR	Lower	Upper	*p*
Age (year)	1.03	0.99	1.06	0.112	0.97	0.94	1.01	0.106
Diabetic complications	1.77	0.68	4.59	0.242				
Neuropathy	1.78	0.83	3.85	0.140	1.39	0.51	3.34	0.558
Retinopathy	1.1	0.47	2.56	0.834				
Cardiovascular disease	2.58	1.15	5.77	0.021	3.25	1.12	6.75	0.028
Nephropathy	1.71	0.781	3.76	0.180	1.34	0.51	3.55	0.465
Peripheral vascular disease	1.73	0.62	4.78	0.294				
Cerebrovascular disease	4.63	0.28	75.91	0.283				
BMI	1.01	0.95	1.06	0.836				
Smoking	1.45	0.57	3.71	0.435				
Hypertension	0.80	0.37	1.71	0.558				
Hyperlipidemia	1.15	0.54	2.46	0.722				

BMI: body mass index; OR: odds ratio.

**Table 4:** Laboratory evaluations of the patients.

	T1DM (*n* = 33)	T2DM (*n* = 149)	*p*	Patients with EPI (*n* = 33)	Patients Without EPI (*n* = 149)	*p*
FE-1 (μg/g)	335 ± 138	360.8 ± 144.1	0.350	116.3 ± 55.4	409.2 ± 93.2	<0.001
Amilase (U/L)	52.8 ± 21.7	68.8 ± 31.9	0.070	64 ± 25.3	66.2 ± 32.1	0.710
Lipase (U/L)	25.7 ± 14.5	43.3 ± 42.7	0.021	37.9 ± 26.3	40.5 ± 42.1	0.730
ALP (U/L)	79.5 ± 29.2	81.5 ± 40.1	0.790	83.4 ± 24.07	80.6 ± 40.8	0.700
FPG (mg/dL)	154.5 ± 86.9	153.2 ± 64.3	0.930	139.4 ± 61.4	156.6 ± 70	0.190
PPG (mg/dL)	239.7 ± 122.7	210.4 ± 79.6	0.190	231.5 ± 99.9	212.2 ± 86.7	0.260
HbA1c (%)	9.13 ± 1.9	8.4 ± 1.8	0.051	8.9 ± 2.1	8.4 ± 1.8	0.160
C-peptide (pmol/mL)	0.71 ± 2.29	3 ± 2.1	<0.001	2.8 ± 3.0	2.5 ± 2.1	0.600
AST (U/L)	17.5 ± 5.3	18.8 ± 6.8	0.290	19.9 ± 8.2	18.3 ± 6.1	0.200
ALT (U/L)	16.3 ± 7.1	20.4 ± 10.7	0.036	20.3 ± 8.4	19.5 ± 10.6	0.710
GGT (U/L)	17.6 ± 17.8	29.5 ± 23.5	0.007	29.8 ± 27.9	26.8 ± 21.8	0.490
Creatinine (mg/dL)	0.69 ± 0.21	1.16 ± 1	<0.001	1.39 ± 1.49	1.0 ± 0.73	0.150
BUN (mg/dL)	12.5 ± 5.4	21.5 ± 15.6	0.010	23.2 ± 20.6	19.1 ± 13.1	0.270
Calcium (mg/dL)	9.6 ± 1.4	9.4 ± 0.5	0.210	9.2 ± 0.7	9.5 ± 0.7	0.043
Phosphorus (mg/dL)	3.5 ± 0.5	3.7 ± 0.7	0.190	3.7 ± 0.84	3.6 ± 0.6	0.510
25-OH Vit D3 (ng/mL)	22.4 ± 12.2	22.4 ± 14.5	0.990	22.8 ± 9.6	22.3 ± 14.9	0.860
LDL (mg/dL)	117.68 ± 26.54	107.19 ± 33.107	0.150	104.93 ± 35.82	110.86 ± 30.74	0.380
HDL (mg/dL)	52.59 ± 13.28	42.15 ± 14.20	0.002	41.46 ± 11.77	45.17 ± 15.32	0.260
TG (mg/dL)	123.4 ± 93.83	176.16 ± 99.84	0.033	166.68 ± 95.27	166.30 ± 102.53	0.980

T1DM: type 1 diabetes mellitus; T2DM: type 2 diabetes mellitus; EPI: exocrine pancreatic insufficiency; FE-1: fecal elastase-1; ALP: alkaline phosphatase; FPG: fasting plasma glucose; PPG: postprandial plasma glucose; HbA1c: hemoglobin A1c; AST: aspartate transaminase; ALT: alanine transaminase; GGT: gamma glutamyl transferase; BUN: blood urea nitrogen; 25-OH Vit D3: 25-hydroxy vitamin D3; LDL: Low-Density Lipoprotein Cholesterol; HDL: High-Density Lipoprotein Cholesterol; TG: Triglycerides.

**Table 5:** Comparison of type 2 diabetes patients according to the presence of insulin therapy.

	Patients with EPI	Patients Without EPI	Total	*p*
Insulin (+)	23	80	103	
Insulin (−)	3	43	46	
Total	26	123	149	0.020

EPI: exocrine pancreatic insufficiency; Insulin (+): patients taking insulin; Insulin (−): patients not taking insulin.

**Table 6:** IPTW-ATT weighted logistic regression model among patients with T2DM (*n* = 149).

Variable	β	OR	95% CI	*p*
Insulin therapy	2.38	10.76	1.85–62.76	0.008
Age (years)	−0.01	0.99	0.94–1.05	0.785
Sex (male)	−0.04	0.96	0.28–3.34	0.950
BMI (kg/m^2^)	0.06	1.06	0.84–1.34	0.606
HbA1c (%)	−0.05	0.95	0.74–1.22	0.684
Hypertension	−1.05	0.35	0.12–1.04	0.059
Hyperlipidemia	0.03	1.03	0.36–2.92	0.955
Weight (kg)	−0.03	0.97	0.88–1.06	0.504
Height (cm)	0.02	1.02	0.91–1.14	0.740
Cardiovascular disease	1.26	3.52	1.22–10.15	0.020

*p* < 0.05. β: regression coefficient; OR: odds ratio; CI: confidence interval. Propensity scores estimated using logistic regression with baseline covariates: age, sex, BMI, HbA1c, hypertension, hyperlipidemia, weight, height, and cardiovascular disease. HDL-C and LDL-C were excluded due to high missing data rates (42.3% and 36.2%, respectively). Missing values for hypertension (*n* = 12, 8.1%) and hyperlipidemia (*n* = 9, 6.0%) were imputed using the mode.

**Table 7:** Standardized mean differences for baseline covariates before and After IPTW-ATT weighting among T2DM patients (*n* = 149).

Variable	SMD Before Weighting	SMD After Weighting
Age (years)	0.12	0.08
Sex (male)	0.42	0.16
BMI (kg/m^2^)	0.08	0.11
HbA1c (%)	1.00	0.25
Weight (kg)	0.25	0.22
Height (cm)	0.30	0.22
Hypertension	0.15	0.06
Hyperlipidemia	0.37	<0.01
Cardiovascular disease	0.07	0.18

Missing hypertension (8.1%) and hyperlipidemia (6.0%) values were imputed using the mode.

## Data Availability

The data supporting the findings of this study are not publicly available due to privacy and ethical restrictions but are available from the corresponding author on reasonable request.

## Abbreviations

The following abbreviations are used in this manuscript:

T1DM	Type 1 diabetes mellitus
T2DM	Type 2 diabetes mellitus
EPI	Exocrine pancreatic insufficiency
FE-1	Fecal elastase-1
BMI	Body mass index
ALT	Alanine aminotransferase
AST	Aspartate aminotransferase
ALP	Alkaline phosphatase
GGT	Gamma-glutamyl transferase
BUN	Blood urea nitrogen
LDL-C	Low-density lipoprotein cholesterol
HDL-C	High-density lipoprotein cholesterol
TG	Triglyceride
IPTW	Inverse probability of treatment weighting
ATT	Average treatment effect on the treated
AUC	Area under the curve
OAD	Oral antidiabetic drug(s)
PERT	Pancreatic Enzyme Replacement Therapy
